# Comparison Study of Cytotoxicity of Bare and Functionalized Zinc Oxide Nanoparticles

**DOI:** 10.3390/ijms22179529

**Published:** 2021-09-02

**Authors:** Anna Król-Górniak, Katarzyna Rafińska, Fernanda Monedeiro, Paweł Pomastowski, Bogusław Buszewski

**Affiliations:** 1 Centre for Modern Interdisciplinary Technologies, Nicolaus Copernicus University in Torun, 4 Wileńska Str., 87-100 Torun, Poland; annkrol18@gmail.com (A.K.-G.); katrafinska@gmail.com (K.R.); fernandamonedeiro@hotmail.com (F.M.); pomastowski.pawel@gmail.com (P.P.); 2 Environmental Chemistry and Bioanalytics, Faculty of Chemistry, Nicolaus Copernicus University in Torun, 7 Gagarina Str., 87-100 Torun, Poland

**Keywords:** zinc oxide nanoparticles, organic surface deposit, cytotoxicity assay, photocatalytic activity, ROS generation, toxicity mechanism

## Abstract

In this paper, a study of the cytotoxicity of bare and functionalized zinc oxide nanoparticles (ZnO NPs) is presented. The functionalized ZnO NPs were obtained by various types of biological methods including microbiological (intra- and extracellular with *Lactobacillus paracasei* strain), phytochemical (*Medicago sativa* plant extract) and biochemical (ovalbumin from egg white protein) synthesis. As a control, the bare ZnO NPs gained by chemical synthesis (commercially available) were tested. The cytotoxicity was measured through the use of (3-(4,5-dimethyl-2-thiazolyl)-2,5-diphenyl-2H-tetrazolium bromide (MTT) dye as well as lactate dehydrogenase (LDH) assays against murine fibroblast L929 and Caco-2 cell lines. As a complementary method, scanning electron microscopy (SEM) was performed to assess the morphology of the tested cells after treatment with ZnO NPs. The microscopic data confirmed the occurrence of apoptotic blebbing and loss of membrane permeability after the administration of all ZnO NPs. The reactive oxygen species (ROS) concentration during the cell lines’ exposure to ZnO NPs was measured fluorometrically. Additionally, the photocatalytic degradation of methylene blue (MB) dye in the different light conditions, as well as the antioxidant activity of bare and functionalized ZnO NPs, is also reported. The addition of all types of tested ZnO NPs to methylene blue resulted in enhanced rates of photo-degradation in the presence of both types of irradiation, but the application of UV light resulted in higher photocatalytic activity of ZnO NPs. Furthermore, bare (chemically synthetized) NPs have been recognized as the strongest photocatalysts. In the context of the obtained results, a mechanism underlying the toxicity of bio-ZnO NPs, including (a) the generation of reactive oxygen species and (b) the induction of apoptosis, is proposed.

## 1. Introduction

Currently, zinc oxide is one of the main pillars of research in the field of nanotechnology and medicine, mainly due to its high applicability—it is widely used in many bactericidal formulations (such as ointments for controlling eczema) or in the cosmetic industry (in UV rays protection creams) [1,2]. The nanomaterials of zinc oxide, including bare nanoparticles (ZnO NPs), but also those functionalized with various types of surface modifications, attract special attention [3,4]. The concept of functionalization (inorganic core and organic deposition) is well known from the chromatographic sciences (the designing of stationary phases with specific modifications) and can be successfully applied to the nanotechnology field [5,6]. Some research groups have proposed the addition of polyethylene glycols (PEGs), which are known for their biocompatibility and biodegradation properties [7,8]. Another widely used type of functionalization is silica coating—silica is the most common material applied in analytical chemistry as column packing for liquid chromatography [5,6], but the thin film of silica can be also used in nano deposition. It seems clear that the surface chemistry of ZnO NPs might be a decisive factor in describing the cell–nanoparticle interactions in in vitro studies. According to data in the literature, functionalized ZnO NPs consisting of an inorganic metal oxide core and organic surface deposition represent a new class of nanomaterials. Furthermore, they exhibit improved properties such as, e.g., stability, biocompatibility and toxicity, in comparison with native nanoparticles. However, not all types of the ZnO NPs’ surface manipulation will result in the enhancement of such attributes. As an example, Yin et al. [9] modified ZnO NPs with a SiO_2_ coating and performed a toxicity study on human lymphoblastoid cells. The experimental data proved that, regardless of the surface modification, the toxicity of bare and functionalized NPs was the same. Recently, a new approach based on the biological production of ZnO NPs was able to be suggested. Based on our latest experience, ZnO NPs can be described as functionalized due to the specific organic deposit on their surface, which comes from the bacterial biomass, for example, the probiotic *Lactobacillus paracasei* strain [10], plant extracts, e.g., *Medicago sativa* [11], or proteins, e.g., egg white protein [12], that is used in the synthesis process. The presence of characteristic organic residuals of natural origin on bio-ZnO NPs significantly increases their luminescence and antimicrobial properties [10,11,12,13]. This subject led our research group to the main motivation of the experiments presented in this paper—the interest in biologically synthetized ZnO NPs as a potentially safe agent for the treatment of oral and external bacterial infections.

Despite many potential medical applications of bio-ZnO NPs, it is necessary to consider the fact that each antimicrobial agent can be risky for human health because of its potential to reach any organ or tissue [4,14,15]. Nano-ZnO might have an impact on the loss of membrane integrity, the decrease in cells viability or even the activation of apoptosis [16]. The mechanisms underlying the nano-toxicity have been studied intensively, but there are still many questions and doubts. One of the possible toxicity mechanisms is the generation of reactive oxygen species (ROS) in cells after the nanomaterials’ treatment [17,18]. The production of radicals by nano-ZnO is strongly related to the catalytic properties of this material. Zinc oxide is a wide-bandgap (3.3 eV) semiconductor and it is able to absorb the UV and other higher energy radiations, producing a hole in the valence band and a free electron in the conduction band [19]. The reactions of holes and/or electrons in ZnO NPs can further produce another activated reactive oxygen species [19,20]. The redox reactions caused by the photoinduced electrons (e^−^) and holes (h^+^) enhance the nano–ZnO photocatalytic activity [21]. The photocatalysis process is commonly used on an industrial scale for the degradation of dyes and pollutants in environmental samples, e.g., water [21,22,23,24]. 

On the other hand, nanomaterials can also show antioxidant activity, which means protecting cells from the damaging effects of reactive oxygen species [4,25,26,27,28]. This can be explained by the transfer of electron density located in oxygen to the odd electron located in the outer orbits of oxygen in OH^•^ and O_2_^•−^ radicals [17,29]. The totally different mode of nano-ZnO action might be associated with the concentration and colloidal stability of the used nanoparticles. Baskar et al. [26] revealed that the antioxidant activity of ZnO NPs followed a decreasing order with increasing concentrations of NP treatment. A similar result was described by Zafar with colleagues [28], who reported that, at lower concentrations, ZnO NPs showed increased 2,2-diphenyl-1-picrylhydrazyl (DPPH) activity in comparison with the higher content of nanoparticles. Moreover, according to [30], with the increasing of the concentration of ZnO NPs, the content of glutathione (GSH) in adipocyte cells also increased. Glutathione is a major antioxidant that can help prevent this process through the removal of ROS. With higher concentrations of ZnO NPs, the level of reactive oxygen species (ROS) was also increased. These results suggest the hypothesis that higher concentrations of nanomaterials exhibit pro-oxidative activity, while, at lower concentrations, the nanoparticles act as antioxidant agents [26,28,30].

According to the Food and Drug Administration (FDA) regulations, physicochemical characterization, assessment of the size distribution, in vitro and in vivo toxicology studies, and the evaluation of photocatalytic properties, are recommended for the safety assessment of nano-ingredients [31]. Furthermore, the choice of the appropriate assay is important for the accurate assessment of NPs’ cytotoxicity. Although there are clear guidelines for nanomaterials against cell lines, there is no consensus on the exact nano-ZnO toxicity mechanism. So far, the basic mechanism of nanotoxicity related to the size and concentration of NPs has been proposed. Valdiglesias with colleagues [32] tested the cytotoxic effect of ZnO NPs of around 100 nm in size on human SH-SY5Y neuronal cells with the MTT assay—the viability of the neuronal cells depended on the NPs’ concentration. As for the second mechanism, the release of toxic zinc ions [33,34] or ROS production was suggested. Although the use of the ZnO NPs as photocatalysts is a great opportunity, it is important to remember that all of ROS might be responsible for lipid peroxidation, DNA damage or even the activation of apoptosis [18]. Punnoose et al. [35] demonstrated that the ZnO NP sample with the higher photocatalytic activity displayed around 1.5-fold stronger cytotoxic effect on the Hut-78 lymphoma T cell line. 

However, despite numerous literature reports, the nanotoxicity mechanism is ambiguous and the specific determination of its foundations is still a challenge. Therefore, the concern for the correlation between photocatalytic activity and cytotoxicity of ZnO NPs seems to be crucial. The translation of biological and catalytic properties of ZnO to applied medicine is a novel approach and will allow the successful design of medical products such as, e.g., bedsore ointments.

Taking the above into consideration, a novel approach for evaluating the biological effect of synthetized ZnO NPs, based on complementary and interdisciplinary methods, is proposed. The present research compares the toxic potential of bare and functionalized ZnO NPs during biological synthesis via microbial (intra- and extracellular), plant-based and biochemical approaches, respectively. Cytotoxicity tests were performed on the Caco-2 and murine fibroblasts L929 cell lines using lactate dehydrogenase (LDH) and tetrazolium dye (MTT) assays. Based on the experimental data, the factors influencing the ZnO nanotoxicity (size, concentration, origin, and the presence of specific organic deposits on the surface) are demonstrated. Furthermore, the tight relationship between the oxidative potential of ZnO NPs (photocatalytic and antioxidant activity) with the cytotoxicity is shown. The described unique attributes of functionalized ZnO NPs indicate their potential for further application in medical fields. Finally, the results of the performed study support the proposed ZnO NPs’ nanotoxicity mechanism.

## 2. Results and Discussion

### 2.1. Antioxidant Activity of ZnO NPs

**Figure 1:**
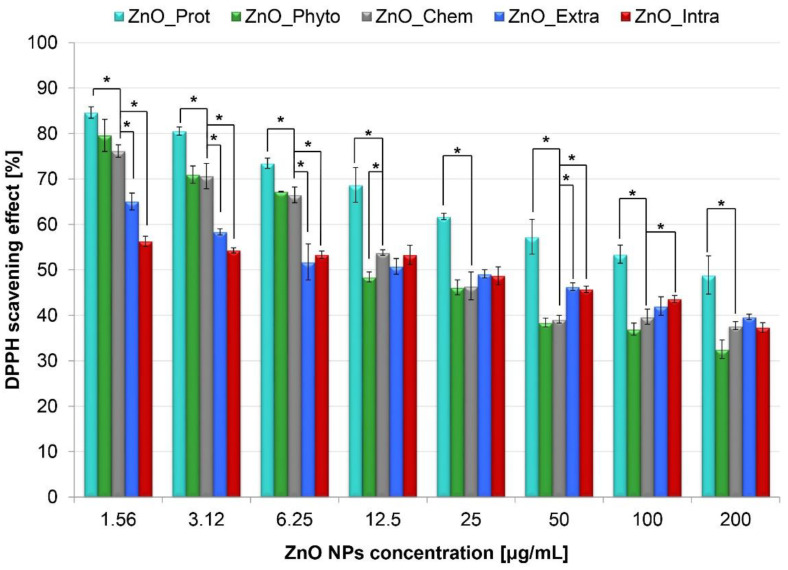
Antioxidant activity (%) of bare and functionalized ZnO NPs (at 200, 100, 50, 25, 12.5, 6.25, 3.12 and 1.56 µg/mL concentrations). The values are expressed as mean ± SD values of three independent experiments (*n* = 3); * *p* < 0.001 compared to bare ZnO NPs (ANOVA).

In Figure 1, the DPPH scavenging activity of bare and functionalized ZnO NPs is presented. The obtained data indicate that the antioxidant activity of ZnO NPs depends on their concentration. At 200 μg/mL concentration, biochemically synthetized ZnO NPs (ZnO_Prot) have the highest ability to scavenge the DPPH radical (48.87 ± 4.21%), whereas the nanoparticles formed by *M. sativa* plant extract (ZnO_Phyto) and by *L. paracasei* LB3 cells (ZnO_Intra) exhibit the lowest antioxidant activity (32.54 ± 2.02 and 37.29 ± 1.07%, respectively). With the decrease in the ZnO NPs’ concentration, their scavenging activity increases to 84.62 ± 1.25% (ZnO_Prot), 79.59 ± 3.54% (ZnO_Phyto), 76.16 ± 1.37% (ZnO_Chem), 65.05 ± 1.85% (ZnO_Extra) and 56.31 ± 1.21% (ZnO_Intra) at 1.56 μg/mL concentration level, respectively (Figure 1). Analysis of variance (ANOVA) between the tested groups revealed the significant influence of the studied concentrations on the DPPH scavenging effect for the bio-ZnO NPs (*p*-value < 0.001) as compared to bare ZnO NPs.

The greatest increase in the antioxidant activity was observed for plant-based nano-ZnO (ZnO_Phyto). The higher ability of two ZnO NPs types (ZnO_Phyto and ZnO_Prot) to scavenge the DPPH radical might be related to the presence of biologically active groups on the surface of nanoparticles. As shown in the previous study [11], *Medicago sativa*, used for the biosynthesis, is a material that is rich in flavonoids, saponins or phenolic compounds. All of them have free radical scavenging abilities [36]. Interestingly, the interaction of flavonoids, such as zinc, with Me^2+^ may enhance the antioxidant properties of the flavonoids [37]. During the biosynthesis of ZnO NPs with *M. sativa* (ZnO_Phyto), an increase in antioxidant activity was observed in comparison with a crude extract [11]. This is in a good correlation with the data obtained in present study. The next ZnO NPs with good antiradical properties were obtained from egg white protein, known as ovalbumin [12]. The data in the literature point out that proteins and peptides derived from egg white produce a good antioxidant effect [37,38,39]. Furthermore, the complexes of zinc with peptides [40] or amino acids (e.g., histidine) [41,42] were proven to have boosted the antioxidant properties.

### 2.2. Photocatalytic Degradation of Methylene Blue (MB) by ZnO NPs

**Figure 2:**
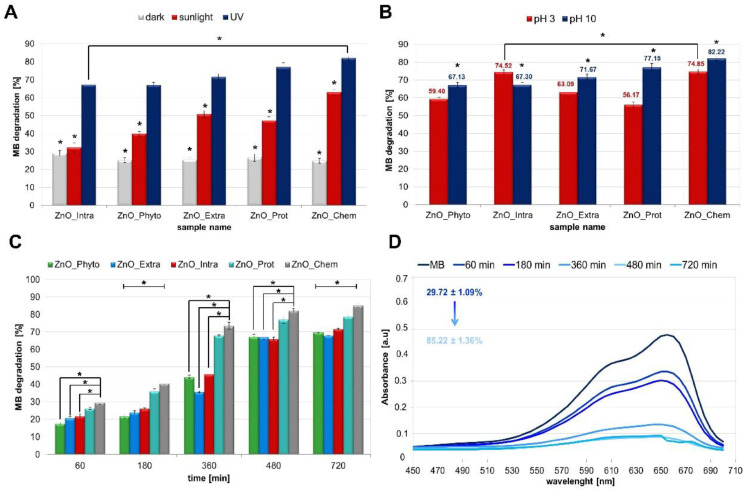
Photocatalytic activity of bare and functionalized ZnO NPs toward methylene blue (MB). The efficiency of MB photocatalytic degradation (%) (**A**) in different light conditions (dark, sunlight and UV irradiation), and (**B**) for different pH values (pH 3 and 10); (**C**) MB degradation time-dependency at pH 10 under UV irradiation; (**D**) UV-vis plot of MB in the presence of ZnO_Chem at pH 10 under UV irradiation. All ZnO NPs were at 1000 μg/mL concentration. The values are expressed as mean ± SD values of three independent experiments (*n* = 3); * *p* < 0.001 compared to bare ZnO NPs (ANOVA).

The bare and functionalized ZnO NPs were used as a photocatalyst for the degradation of methylene blue dye under different light conditions (dark, sunlight and UV light at λ = 365 nm; Figure 2A). After 8 h of photocatalysis, the degradation of MB in the dark was the slowest in comparison with the light conditions (sunlight and UV irradiation). Moreover, for all types of tested nanoparticles, the percentage of MB degradation was in the range of 24.88 ± 1.3–29.12 ± 1.4% (dark). In the sunlight, at the same time (8 h), the MB degradation was found to be at the 32.37 ± 2.48–63.04 ± 1.64% level. The highest degradation of methylene blue was observed under UV irradiation and the percentage of this varied from 67.13 ± 1.42 to 82.22 ± 1.1%. The greater efficiency of photocatalysis after UV irradiation finds its explanation in the semiconductor properties of zinc oxide. ZnO has a band gap of 3.3 eV, which corresponds to the emission in the UV region [19]. When the photon energy (*hν*) is equal or exceeds the band gap of the photocatalyst, the photon is absorbed. Next, the electron from the VB is excited to the CB—at the same time, the positive charge holes in the valance band are created. Then, the electron and hole pairs might take part in a series of redox reactions, leading to the formation of reactive oxygen species [20,21,31].

In addition to factors such as the type of irradiation or the type of nanoparticles used in photocatalysis, it is also important to consider the effect of pH [43,44]. In order to investigate the influence of pH on bare and functionalized ZnO NPs’ photocatalytic activity, the degradation of MB dye was studied in acidic and alkaline conditions (Figure 2B). It was observed that the decolorization of methylene blue is strongly dependent on the pH of the solution, which plays an important role in photocatalytic degradation. From the chart (Figure 2B), it can be seen that about 56% and 77% of MB dye was degraded after 480 min illumination by the ZnO_Prot NPs at pH = 3 and pH = 10, respectively. This tendency was visible for almost all tested nanoparticles except for the ZnO_Intra NPs, which show a slightly higher ability to degrade the dye at pH = 3 (Figure 2B). The explanation of this phenomenon can be the fact that the alkaline pH value could provide a higher concentration of hydroxyl ions, which are able to react with holes and form hydroxyl radicals. In consequence, the photocatalytic degradation of methylene blue is enhanced [1,2]. Therefore, the presence of UV-irradiation and the pH value of 10 have been recognized as the most efficient conditions in terms of photocatalysis speed (Figure 2C) and were, therefore, chosen as the final experimental conditions. ANOVA on the tested experimental conditions revealed significant results (*p*-value < 0.001) (showed in Figure 2A–C), indicating that the type of tested ZnO NPs had a significant influence on the observed MB degradation, under all of the studied conditions. Additionally, Dunnett’s post hoc test was performed in order to compare the results from each type of functionalized ZnO NPs in relation to the bare (ZnO_Chem) NPs. In addition, the average percentages of MB degradation, observed for conditions of dark, sunlight and UV light, were significantly different in all ZnO NPs (*p* < 0.001). The results of the post hoc assay are summarized in the Supplementary Material (Tables S1 and S2) and are presented in the form of asterisks in Figure 2.

Methylene blue dye shows a prominent peak at λ = 665 nm, as shown in Figure 2D. The peak intensity decreases gradually with the addition of ZnO NPs under UV irradiation and shows an increase in the MB degradation from 29.72 ± 1.09 to 85.22 ± 1.36% within 720 min. To sum up, among all tested nanoparticles, the bare chemically obtained NPs (ZnO_Chem) are considered as the most efficient photocatalysts. Those data are the opposite of the DPPH test results—the highest antioxidant activity was shown by the nanoparticles that were also the weakest photocatalysts (ZnO_Phyto). ZnO NPs synthetized by *M. sativa* aqueous extract showed a DPPH radical scavenging effect at the 79.59 ± 3.54% level (1.56 μg/mL concentration), while the higher concentration (1000 μg/mL) of the tested nanoparticles allowed degradation only with 17.53 ± 0.77% of dye. This strongly supports the hypothesis that higher concentrations of nanomaterials exhibit pro-oxidative activity, while lower concentrations of nanoparticles act as antioxidant agents [29,45]. Furthermore, it is clear from the experimental data and our previous works [10,11,12,13] that the differences between the bare and functionalized ZnO NPs photocatalytic activity may be related to the size of the nanoparticles. Bare NPs (ZnO_Chem) were recognized as the strongest photocatalysts—the size of this nanomaterial was about 100 nm. The remaining functionalized NPs (ZnO_Prot, ZnO_Intra, ZnO_Phyto, ZnO_Extra) were smaller, at a size of 40, 16.7, 13.9 and 13.7 nm, respectively [10,11,12,13]. Thus, it can be stated that photocatalytic activity increases with the increase in the nano-ZnO particle size. The work of Kusiak-Nejman et al. [46] confirmed that the ZnO NPs’ photoactivity under UV light increases mainly with the increase in the ZnO particle size. The highest phenol degradation was found for a ZnO sample with an average particle size of 71 nm [46]. Murakami et al. [47] prepared titanium(IV) oxide (TiO_2_) NPs for the photocatalytic degradation of acetaldehyde. They found a size of about 40 nm to be optimal for the effectiveness of the photocatalysis process, mainly due to the optimized balance between efficient separation of redox sites and large specific surface area [47]. 

According to the data in the literature [19,48,49], the dyes’ photo-degradation might be related to the generation of electron–hole pairs. The mechanism underlying the process described in this study is discussed later.

### 2.3. Cytotoxicity of ZnO NPs

To examine the cytotoxicity effect of bare and functionalized ZnO NPs in vitro, two cell lines—murine fibroblast L929 and human epithelial colorectal adenocarcinoma Caco-2—were chosen. Caco-2 cells show many morphological and biochemical similarities to intestinal cells, or enterocytes. In addition, this type of cell line is commonly used in the pharmaceutical industry as an in vitro model of the human small intestine mucosa to predict the absorption of orally administered drugs [50,51]. Moreover, Caco-2 cells were used as model to assess the toxicity of ZnO NPs in many studies [52,53,54,55]. The application of mouse fibroblast L929 cells is most frequently undertaken to evaluate cytotoxicity and may represent a sufficient in vitro screening model for skin formulations [56,57,58]. Furthermore, the cytotoxicity assay with mouse fibroblasts L929 is in compliance with ISO 10993-5 standards, and is often used for comparative studies of different types of nanoparticles [59].

The anti-proliferative effect was determined using two colorimetric assays—3–(4,5–dimethylthiazol–2–yl)–2,5-diphenyl tetrazolium bromide (MTT) and lactate dehydrogenase (LDH). The first of these, the MTT test, is based on the ability of mitochondrial dehydrogenase enzyme to convert yellow tetrazolium dye into formazan crystal. The rate of formazan crystal formation is directly proportional to cell viability; the untreated (positive) control is set to 100% viability [60,61]. In the LDH assay protocol, lactate dehydrogenase, as a soluble cytosolic enzyme, is released into the culture medium following the loss of membrane integrity. Then, LDH activity can be used as an indicator of cell membrane integrity and serve as a general means to assess cell viability by measuring plasma membrane permeability [62].

**Figure 3:**
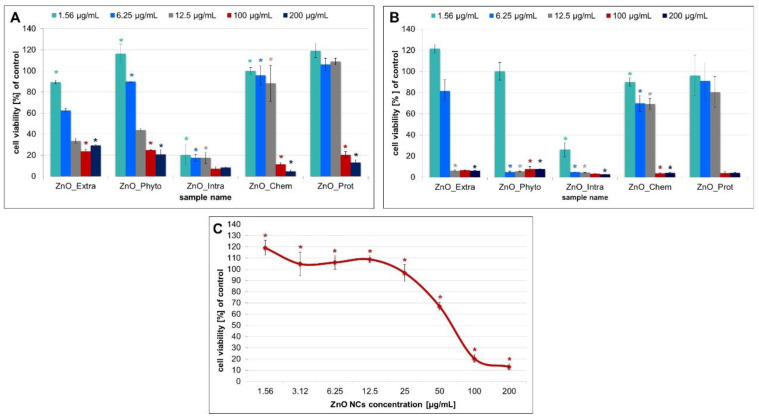
The MTT assay results and effects of indicated concentrations of bare and functionalized ZnO NPs on the (**A**) Caco-2 cells and (**B**) L929 cell viability. (**C**) the effect of different concentrations of biochemically synthetized ZnO NPs against the Caco-2 cell line. The values are expressed as mean ± SD values of three independent experiments (*n* = 3); * *p* < 0.001 compared to bare ZnO NPs (ANOVA).

As shown in Figure 3A,B, the MTT results demonstrated that, for both types of cell lines, higher concentrations of ZnO NPs generated more serious cytotoxicity. At the lowest concentration of 1.56 μg/mL, intracellularly synthetized ZnO NPs (ZnO_Intra) were able to reduce both Caco-2 and L929 cells’ viability to 20.28 ± 9.54 and 26.09 ± 6.74%, respectively. On the other hand, the biochemically synthetized NPs (ZnO_Prot) turned out to be the least toxic to cells—the concentration of 50 μg/mL might be considered as an IC_50_. At the highest concentration (100 and 200 μg/mL) (Figure 3C), all types of tested nanoparticles significantly reduced the cells’ viability, while the bare and functionalized ZnO NPs were significantly more toxic to the fibroblasts L929 (Figure 3B). The work of Valdiglesias et al. [32] also showed the concentration-dependent toxicity of nano-ZnO—significance was obtained from 25 μg/mL for all of the treatments. Despite the crucial role of the ZnO NPs’ dose, the size of the nanoparticles used should be also taken into consideration. Many papers indicate the influence of the nano-ZnO size on its further toxicity. Kang et al. [55] tested ZnO NPs at different sizes (26, 62 and 90 nm) against Caco-2 cells and observed that 26-nm ZnO NPs exhibited the highest toxicity. The different toxic effects of ZnO_Intra and ZnO_Prot NPs can be also explained by their various sizes—as mentioned above, the size of biochemically (ZnO_Prot) synthetized NPs was about 41 nm [12], whereas the ZnO_Intra NPs were smaller, at a size of 16.7 nm [10]. Furthermore, biologically synthetized NPs are functionalized by the specific organic deposit on their surface—the presence of organic residues was confirmed by the LDI-MS method in our previous study [13]. It was proven that the organic deposit plays an important role in the antibacterial action of nanomaterials. Silver [27] and zinc oxide NPs [13], naturally coated by microbial compounds, exhibit higher antibacterial effects compared with chemically synthetized nanomaterials. Intriguingly, the presence of the surface deposit might affect the cell–NPs interaction, and consequently, the intended bio-application of the obtained nanoparticles. As shown in the present paper, the extracellularly synthetized ZnO NPs, with organic deposits on their surfaces, were less toxic for Caco-2 cells than the chemically obtained NPs (29.26 ± 1.15% and 4.81 ± 1.57%, respectively, at 200 μg/mL concentration).

The MTT results presented in this paper are also in good correlation with the data from the photocatalytic study. The highest tested concentration of ZnO NPs resulted in a significant reduction in the number of live cells (Figure 3) as well as in the degradation of MB dye (Figure 2A). The photocatalytic activity of nano-ZnO is connected to the toxic oxygen species [18,20,21]. In consequence, the toxicity of effect is enhanced.

**Figure 4:**
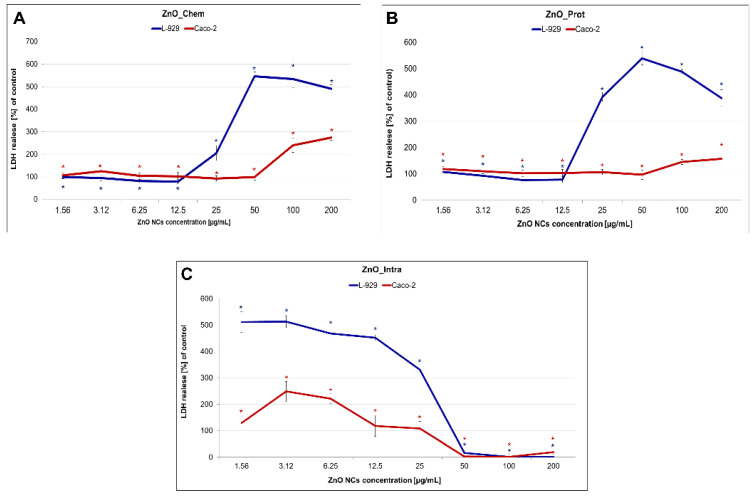
LDH leakage level of Caco-2 and L929 cells treated with different concentrations of (**A**) chemically, (**B**) biochemically and (**C**) intracellularly synthetized ZnO NPs. The values are expressed as mean ± SD values of three independent experiments (*n* = 3); * *p* < 0.001 (ANOVA).

The decrease in cell viability is very often correlated with apoptosis. Cell death occurred as a result of, e.g., cell membrane damage, and, in consequence, the release of LDH into the extracellular medium took place. In our study, LDH activity was measured to observe the effect of bare and functionalized ZnO NPs on membrane integrity by treating fibroblast L929 and Caco-2 cells. The results of this assay show that the enzyme release depends on the nanoparticles concentration, as shown in Figure 4. The ZnO_Chem and ZnO_Prot NPs exhibited a significant increase in LDH leakage at 25–100 μg/mL for the L929 cell line (Figure 4A,B). On the contrary, the intracellularly synthetized NPs (ZnO_Intra) already caused the release of LDH at the 1.56 µg/mL concentration level (Figure 4C) for both types of tested cells. The lack of leakage of LDH at 50–200 µg/mL was not due to a lack of ZnO_Intra NPs toxicity, but the fact that, at this concentration, the cell number was too small to give the correct results (Figure 4C). The tested nano-ZnO were more toxic against the fibroblast L929 than Caco-2 cell line. The higher sensitivity of murine fibroblasts could be explained by a different expression of specific control mechanisms, which play an important role in the differentiation and the apoptosis of various cells. One such mechanism is MAPKs (mitogen-activated protein kinases) [63]. Krüger et al. [64] showed that the addition of TiO_2_ NPs activated the p38 mitogen-activated protein kinase pathways in Caco-2 cells and, furthermore, did not affect enterocyte differentiation. Another mechanism of cell lines’ resistance might be associated with the induction or inhibition of the membrane transporting proteins. Guarnieri et al. [65] emphasized the correlation between metallic uptake, intracellular localization and cytotoxicity. Brück with colleagues [66] compared two different types of cells and showed that the protein expression patterns of membrane transporters in Caco-2 cells and jejunal cells differed notably. However, a detailed genomic and proteomic study regarding the exact nanotoxicity mechanism of tested nanoparticles is required to confirm the differences between the cell lines used in our study. 

**Figure 5:**
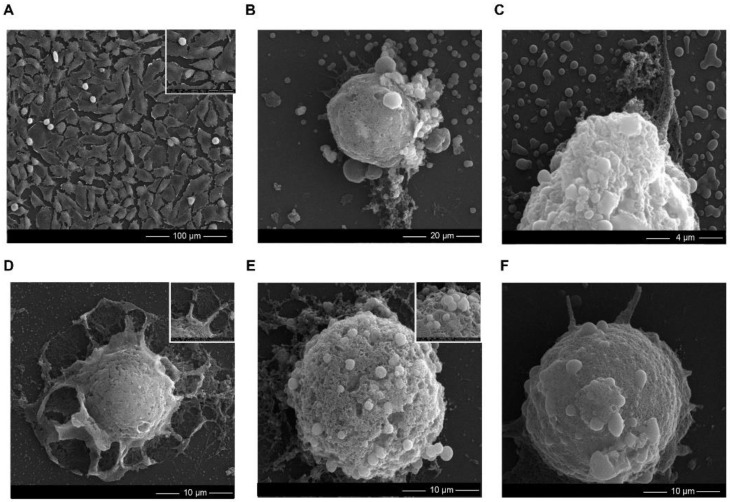
SEM analysis showing the morphology changes in L929 cells after the bare and functionalized ZnO NPs treatement. (**A**) control sample and L929 cells after the (**B**) extracellularly, (**C**) phytochemically, (**D**) intracellularly, (**E**) chemically and (**F**) biochemically synthetized ZnO NPs’ exposure. The concentration of the used ZnO NPs was 6.25 μg/mL.

To observe the changes in murine fibroblast cells’ morphology, scanning electron microscopy (SEM) images, after treatment with 6.25 μg/mL of bare and functionalized ZnO NPs (the concentrations where an effect on the cell growth was noticed), were obtained. According to Figure 5, cells exposed to nano-ZnO differed significantly from the control. The L929 cells treated with the different types of nanoparticles became spherical and specific blebs were formed on their surface (Figure 5B–F). Moreover, the ZnO_Intra NPs caused the destruction of cells such as, e.g., cell membrane collapse (Figure 5D). All of the noticed morphological changes are related to the apoptosis process [67], in which the cell breaks into several vesicles, which are known as apoptotic bodies [68].

**Figure 6:**
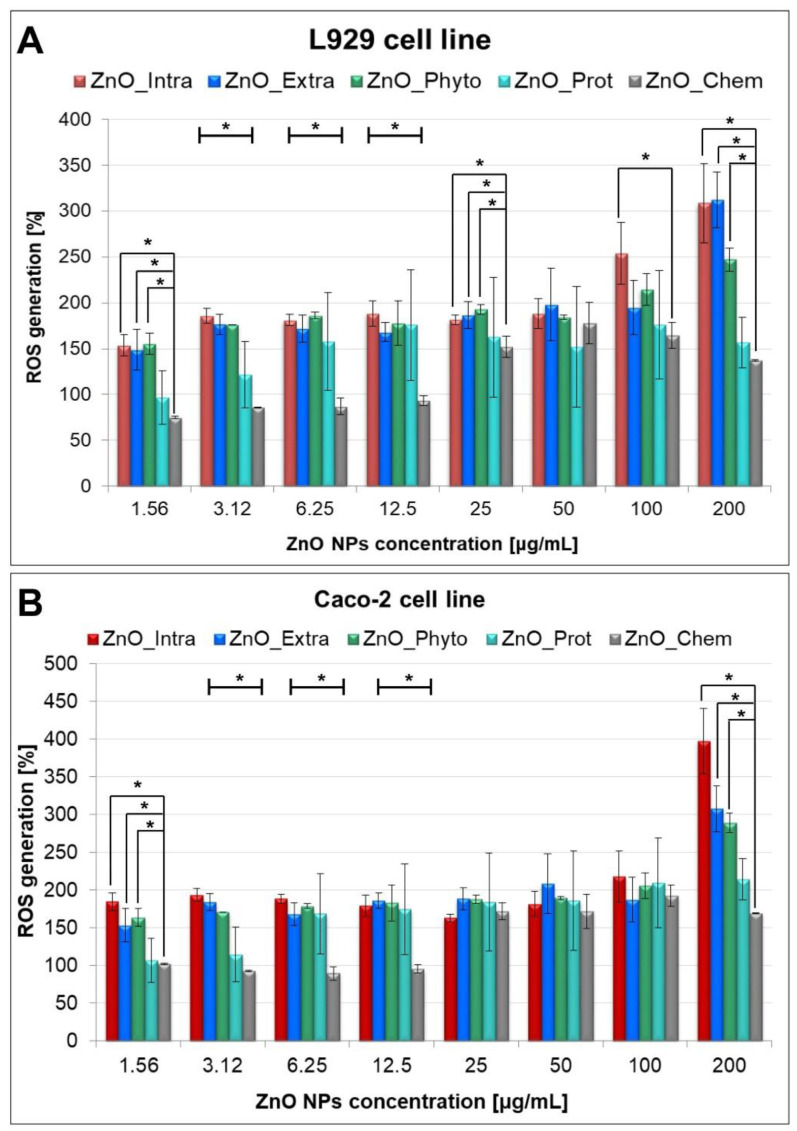
Concentration-dependent ROS generation by bare and functionalized ZnO-NPs in (**A**) L929 and (**B**) Caco-2 cells. The values are expressed as mean ± SD values of three independent experiments (*n* = 3); * *p* < 0.001 compared to bare ZnO NPs (ANOVA).

The interaction of nanoparticles with cells’ surfaces leads to the loss of membrane integrity, but also presents itself in the production of reactive oxygen species (ROS). In order to detect the ROS involved in the MB degradation and the cytotoxicity of the tested bare and functionalized ZnO NPs, a fluorometric intracellular ROS Kit (Sigma-Aldrich, St. Louis, MO, USA) was used (Figure 6). Importantly, the applied kit detects superoxide and hydroxyl radicals in particular. Murine fibroblasts and human epithelium cells exposed to ZnO NPs exhibited a significant concentration-dependent increase in intracellular ROS generation. Figure 6 shows that intracellularly synthetized (ZnO_Intra) NPs cause the highest increase in ROS production—from 153.65 ± 11.52% (at 1.56 µg/mL) to 308.49 ± 43.29% (at 200 µg/mL). The observed tendency may be explained by the fact that, at higher NP concentrations, the higher amount of OH^•^ and O_2_^•−^ radicals production exceeds the defense capability of the cells [69]. Laurent et al. [70] and Popescu et al. [69] observed that treating cell lines with low amounts of ROS increased their proliferative rate, while further increased amounts of radicals resulted in cell death. Oxygen radicals were also found to be produced during the photocatalysis process [21]—the ZnO_Intra NPs were found to degrade the 71.67 ± 0.47% of methylene blue after 12 h and the same type of nanocomposite showed the greatest production of ROS in tested cells. In contrast to the ZnO_Intra NPs, NPs obtained with OVA protein (ZnO_Prot) act as antioxidant agent at the lowest concentration (75.19 ± 29.46% and 106.56 ± 3.65% for L929 and Caco-2 cell lines, respectively). For both types of tested cells, a similar trend was observed in terms of ROS production. It is strongly associated with DPPH assay results (Figure 1) and confirms the thesis that higher concentrations of nano-ZnO promote pro-oxidative activity, while lower concentrations act as antioxidant agents.

In summary, based on the experimental data obtained in this study, it can be concluded that the type of biological synthesis influences the further toxic properties of functionalized ZnO NPs. The phyto- and biochemical synthesis resulted in the nanoparticles having high antioxidant activity toward the DPPH radical. It is well known that both raw sources used for the ZnO NPs production (*Medicago sativa* extract and OVA protein [11,12]) have scavenging ability [36,37,38,39]. Furthermore, the interaction of metal ions (Me^2+^), including zinc, with peptides [40] or flavonoids [37] might enhance the antioxidant properties. Intriguingly, the intracellular synthesis of nano-ZnO using the bacterial biomass [10] turns out to produce the most toxic nanoparticles. It might be related to their size (16.7 nm)—numerous factors, including composition, size and shape, are well known to influence nanotoxicity [71]. The crucial aspect is also the presence of organic deposits on the nanoparticles’ surface, which might be the key in the reduction in ZnO NPs’ toxicity. Experimental data from the actual study confirmed the presence of less cytotoxicity of extracellularly synthetized ZnO NPs [13] with organic constituents on the surface (against Caco-2 cells) compared to the bare, chemically obtained nanoparticles. 

**Figure 7:**
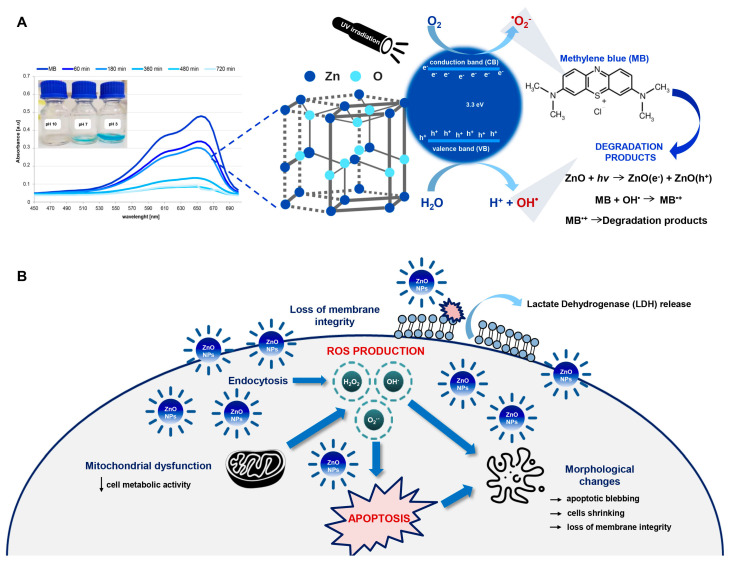
The proposed mechanism of (**A**) photocatalytic degradation of methylene blue and (**B**) cytotoxicity of functionalized ZnO NPs.

According to the data in the literature, it seems that the mechanism of toxicity depends on the aforementioned properties of the nanomaterials (size, concentration, catalytic/luminescence properties or surface characterization and coatings) [3,4,35,46]. However, the exact mechanisms and the material dependence of ZnO nanomaterials’ cytotoxicity is still unclear. Metal oxide nanomaterials are well known to alter the environment around the cells and, thus, induce ROS generation [69,72]. It is also well reported that different mechanisms are proposed for nano-toxicity and one of them includes the production of radicals [4,14,17,18,73]. Based on the experimental results obtained in this study, it can be concluded that the generation of ROS plays a key role in the inhibition of cell growth, the loss of their membrane integrity and, in consequence, the apoptosis process. Moreover, the tight relationship between the harmful impact of the oxygen radical on the living cells and the bio-ZnO NPs’ photocatalytic activity could clearly explain the probable mechanism of bio-ZnO NPs toxicity (Figure 7). During photocatalysis, specific electron–hole pairs are created—the reactions of holes and/or electrons in ZnO NPs lead to the production of reactive oxygen species (Figure 7B). Accordingly, the bio-NPs with greater catalytic properties also exhibited higher toxic impacts on the tested cell lines.

Despite the observed dose-dependent action of ZnO NPs, which is related to greater ROS production, attention should be also paid to another possible nanotoxicity mechanism. The smaller nanoparticles (e.g., ZnO_Intra; about 16.7 nm) have an extraordinary ability to internalize into living cells through different biological mechanisms and reach the nucleus, thereby generating genotoxic damage with the consequent triggering of apoptosis. Mittag et al. [74] investigated the cellular uptake of two differently sized ZnO NPs (<50 nm and <100 nm) against two human intestinal cell lines (Caco-2 and LT97). The outcomes of this study showed that ZnO NPs, at smaller sizes (<50 nm), led to the formation of micronuclei in LT97 cells. Micronucleus analysis is normally performed to examine whether some potentially toxic agent is able to cause chromosome damage [75]. To conclude, the smaller nanoparticles might often exhibit a strong genotoxic activity. Therefore, the undertaking of more detailed studies of ZnO NPs’ genotoxicity seems to be crucial. 

## 3. Materials and Methods

### 3.1. Biological Synthesis and Physicochemical Characterization of ZnO NPs

Four different biological methods of synthesis—phytochemical using *Medicago sativa* plant extract (ZnO_Phyto) [11], microbiological intracellular using *Lactobacillus paracasei* LB3 biomass (ZnO_Intra) [10], microbiological extracellular with *Lactobacillus paracasei* supernatant (ZnO_Extra) [13] and a biochemical approach with ovalbumin (OVA) protein (ZnO_Prot) [12]—were used to obtain the ZnO NPs. All biologically synthetized nanoparticles were functionalized by the presence of the specific organic deposit on their surface. ZnO_Prot, ZnO_Intra, ZnO_Phyto and ZnO_Extra NPs were found to have sizes of 40, 16.7, 13.9 and 13.7 nm, respectively. All further details of the synthesis methods as well as physicochemical characterization of all ZnO NPs are described in our previous papers. 

For the comparison, in all experiments, bare chemically synthetized nano-ZnO (ZnO_Chem) NPs from Sigma-Aldrich, Poznań, Poland (CAS number: 1314-13-2; <110 nm particle size) were used. 

### 3.2. Antioxidant Activity of ZnO NPs

Evaluation of the antioxidant activity of bare and functionalized ZnO NPs was performed using 2,2-diphenyl-1-picrylhydrazyl (DPPH) test [76]. The DPPH radical (Sigma-Aldrich, Steinheim, Germany), at 0.1 M concentration in methanol, was added to different concentrations (200, 100, 50, 25, 12.5, 6.25, 3.12 and 1.56 µg/mL) of each bio-ZnO NP at a ratio of 1:1. All samples were incubated in the dark for 30 min; after the incubation time, the UV-vis absorbance at λ = 517 nm was recorded using a NanoDrop 2000 spectrophotometer (Thermo Scientific, Waltham, MA, USA). The DPPH scavenging activity was calculated using the following equation:

(1) $$AA\%\;=\;\frac{\left(A_{0}\;-\;A_{1}\right)}{A_{0}}\;\times \;100$$

where *A*_0_ and *A*_1_ are absorbance of DPPH and ZnO NPs sample at λ = 517 nm, respectively. All of the experiments were prepared in triplicate.

### 3.3. Photocatalytic Degradation of Methylene Blue (MB) Dye by ZnO NPs

The bare and functionalized ZnO NPs were studied in terms of their catalytic properties under sunlight and UV-light (λ = 365 nm), during the degradation of methylene blue (MB) dye, according to a procedure adapted from [77]. The ZnO NPs, at 1000 µg/mL concentration, were added to the dye solutions (Aqua-Med, Łódź, Poland) at 0.015 mg/mL concentration and pH = 3 and 10. The mixture was magnetically stirred in dark conditions in order to maintain the absorption–desorption equilibrium. After the incubation, the suspension of MB dye and ZnO NPs was subjected to solar light and UV irradiation, respectively. The UV-vis spectra and absorption maxima (λ = 665 nm) were registered at fixed intervals using Varioskan TM LUX multimode microplate reader (Thermo Fisher Scientific, Waltham, MA, USA). The experiment was performed in triplicate, and the photocatalytic degradation was calculated using Equation (1), where A_0_ and A_1_ are the absorbance of MB and MB with ZnO NPs sample at λ = 665 nm, respectively. As a control, the degradation of MB dye was carried out in dark conditions. 

### 3.4. Cytotoxicity of ZnO NPs

The L929 normal mouse fibroblast cells and Caco-2 from human colon were obtained from the European Collection of Authenticated Cell Cultures. The cells were cultured as adherent monolayers in Dulbecco’s modified Eagle’s medium supplemented with 10% fetal bovine serum, 100 U/mL penicillin, and 100 μg/mL streptomycin. Cells were passaged using 0.25% trypsin/EDTA every 3–4 days.

#### 3.4.1. MTT, LDH Release and Intracellular ROS Assays

For MTT, LDH release and intracellular ROS assays, cells were cultured on 96-well plates at 2 × 10^5^ cells/mL and incubated under 5% CO_2_ at 37 °C for 24 h. When cells were adherent, ZnO NPs were added and incubated for another 24 h. In all cell experiments, the L929 and Caco-2 cells without ZnO NPs were used as controls. Then, 10 μL of Thiazolyl Blue Tetrazolium Bromide (MTT) solution (5 mg/mL in PBS) was added and incubated for 4 h at 37 °C. After incubation, medium from wells was discarded and the crystalline formazan was dissolved in DMSO. Absorbance was measured at λ = 570 nm using a Varioskan TM LUX multimode microplate reader (Thermo Fisher Scientific, Waltham, MA, USA). The results were expressed as percentages relative to the negative control—cells in standard medium without ZnO NPs that are considered as 100% viable. All experiments were performed in three independent replicates.

The LDH release assay was performed using a commercially available kit from Sigma Aldrich (Lactate Dehydrogenase Activity Assay Kit MAK066) and all samples were prepared according to the manufacturer’s instructions Absorbance was measured at λ = 450 nm using a Varioskan TM LUX multimode microplate reader (Thermo Fisher Scientific, Waltham, MA, USA). Briefly, cultured cells were incubated with bare and functionalized ZnO NPs to induce cytotoxicity and subsequently release lactate dehydrogenase (LDH). The LDH released into the medium was transferred to a new plate and mixed with 50 μL of the Reaction Mixture in each well. The plate was protected from light during the incubation (37 °C). The measurements of absorbance were taken each 5 min until the value of the most active sample was higher than the value of the highest standard in the standard curve (12.5 nmole/well). The results are presented as percentages of activity in comparison to control. All samples were run in the three independent replicates.

For reactive oxygen species measurements, the Fluorometric Intracellular ROS kit (MAK144), from Sigma-Aldrich, was used. All samples were prepared according to the manufacturer’s instructions. To induce ROS, the cells, after bare and functionalized ZnO NPs treatment, were incubated in 96-well plates in a 5% CO_2_, 37 °C incubator for 24 h. After the incubation time, 100 μL of Master Reaction Mix were added to each well and the cells were incubated again in a 5% CO_2_, 37 °C incubator for 30 min. After that, the fluorescence intensities (λ_ex_ = 540/λ_em_ = 570 nm) were measured using a Varioskan TM LUX multimode microplate reader (Thermo Fisher Scientific, Waltham, MA, USA). Each experiment was performed in the three independent replicates.

The results of the LDH release and ROS level measurement are presented as percentages of reactive oxygen species in comparison to the control sample, and were calculated according to Kalińska et al. [78] and Lin et al. [79], respectively. 

#### 3.4.2. SEM Analysis

Adherent cells were fixed in 2.5% glutaraldehyde in PBS for 30 min and washed 3 × 5 min times in PBS. The next step was washing 2 × 2 min with dH_2_O, dehydration in 95% EtOH for 1 × 2 min followed by 4 × 5 min in 100% EtOH. In the next step, cells were dehydrated 2 × 10 min with 100% HMDS at room temperature. Slides with cells were fixed on a holder with carbon tape and coated with Au with in a SC7620 Mini Sputter Coater (Quorum Technologies, Lewes, UK). The cells were examined using a Quanta 3D FEG scanning electron microscope/focused ion beam (SEM/FIB).

#### 3.4.3. Statistical Data Analysis

Significant differences among means of the groups were evaluated using one-way analysis of variance (ANOVA). The test was performed employing the software IBM SPSS Statistics v.23. Additionally, Dunnett’s post hoc test was performed in order to compare the results from each type of functionalized ZnO NPs in relation to the bare (ZnO_Chem) NPs. The results of post hoc assay are summarized in the Supplementary Material (Tables S1–S6).

## 4. Conclusions

This study presents, for the first time, the comparative evaluation of the cytotoxicity of bare and functionalized ZnO NPs against L929 murine fibroblasts and Caco-2 cells as model cell lines. The outcomes of this work showed the dependence of ZnO NPs’ concentration and size on their biological properties such as antioxidant, photocatalytic and cytotoxic activity. Moreover, the toxic action of functionalized ZnO NPs can be also connected to the presence of a specific organic deposit on their surface. Intracellularly and chemically synthetized ZnO NPs were considered to be the most toxic agent, while the ZnO NPs obtained with ovalbumin protein exhibited the lowest toxicity toward the tested cells. Intriguingly, cell lines showed different sensitivities to ZnO NP treatment—the murine fibroblast L929 was discovered to be more susceptible than Caco-2 cell lines. Accordingly, the functionalized bio-ZnO NPs might be a promising antibacterial agent when used as an oral treatment rather than in skin formulations. The ROS generation assay and SEM microscopy confirmed the oxidative stress induction and the morphological changes in the cells exposed to the ZnO NPs. Based on the data from our investigation, the bio-ZnO NPs toxicity mechanism was proposed—it is tightly related to the generation of ROS which leads to, e.g., the loss of membrane permeability, mitochondrial dysfunction and, at the highest concentrations, the apoptosis of cells.

## Data Availability

Not applicable.

## Supplementary Materials

The following are available online at https://www.mdpi.com/article/10.3390/ijms22179529/s1.
