# Supplementary material for: Comparison Study of Cytotoxicity of Bare and Functionalized Zinc Oxide Nanoparticles
Source: Int J Mol Sci. 2021 Sep 2;22(17):9529. doi: 10.3390/ijms22179529 (PMC8431566; doi:10.3390/ijms22179529)
Supplement: Supplementary file 1 [file ijms-22-09529-s001.zip › ijms-1343606-supplementary.pdf]

Supplementary Material

Comparison study of cytotoxicity of bare and functionalized zinc oxide nanoparticles

Anna Król-Górniak[1.2]. Katarzyna Rafińska[1.2]. Fernanda Monedeiro[1.2] . Paweł Pomastowski[1] and Bogusław Buszewski[1.2]*

Significant differences among means of the groups were evaluated using one-way analysis of variance (ANOVA). The test was performed employing the software IBM SPSS Statistics v.23. Additionally, Dunnett's post hoc test was performed in order to compare the results from each type of functionalized ZnO NPs in relation to the bare (ZnO_Chem) NPs. The results of post hoc assay are summarized in the Supplementary Material (**Table S1 – S6**).

Table S1 The Dunnett post hoc test for MB degradation at different conditions. Cross correlation between each type of functionalized Zno NPs in comparison to the bare ZnO NPs. The statistically significant correlations ($p < 0.01$) are marked in the red colour

Dependent variable	Tested samples		Significance (p-value)
**dark**	ZnO_Intra	ZnO_Chem	6.93×10^{-8}
	ZnO_Phyto		6.45×10^{-8}
	ZnO_Extra		1.37×10^{-6}
	ZnO_Prot		7.84×10^{-4}
**sunlight**	ZnO_Intra	ZnO_Chem	3.21×10^{-8}
	ZnO_Phyto		3.22×10^{-8}
	ZnO_Extra		8.18×10^{-8}
	ZnO_Prot		3.71×10^{-8}
**UV irradiation**	ZnO_Intra	ZnO_Chem	8.22×10^{-3}
	ZnO_Phyto		9.96×10^{-1}
	ZnO_Extra		9.54×10^{-1}
	ZnO_Prot		2.97×10^{-1}
**pH 3**	ZnO_Intra	ZnO_Chem	2.04×10^{-2}
	ZnO_Phyto		2.15×10^{-1}
	ZnO_Extra		9.97×10^{-1}
	ZnO_Prot		1.00
**pH 10**	ZnO_Intra	ZnO_Chem	4.32×10^{-8}
	ZnO_Phyto		1.92×10^{-7}
	ZnO_Extra		4.80×10^{-5}
	ZnO_Prot		7.36×10^{-6}

Table S2 The Dunnett post hoc test for MB degradation in various time points. Cross correlation between each type of functionalized ZnO NPs in comparison to the bare ZnO NPs. The statistically significant correlations ($p < 0.01$) are marked in the red colour

Dependent variable	Tested samples		Significance (p-value)
**t = 60 min**	ZnO_Intra	ZnO_Chem	9.31×10^{-4}
	ZnO_Phyto		2.34×10^{-5}
	ZnO_Extra		2.55×10^{-4}
	ZnO_Prot		1.01×10^{-1}
**t = 180 min**	ZnO_Intra	ZnO_Chem	9.10×10^{-6}
	ZnO_Phyto		4.18×10^{-7}

	ZnO_Extra		1.78 x 10^{-6}
	ZnO_Prot		3.50 x 10^{-2}
**t = 360 min**	ZnO_Intra	ZnO_Chem	3.02 x 10^{-7}
	ZnO_Phyto		2.39 x 10^{-7}
	ZnO_Extra		4.85 x 10^{-8}
	ZnO_Prot		5.26 x 10^{-2}
**t = 480 min**	ZnO_Intra	ZnO_Chem	1.3 x 10^{-5}
	ZnO_Phyto		3.61 x 10^{-5}
	ZnO_Extra		3.67 x 10^{-5}
	ZnO_Prot		7.78 x 10^{-2}
**t = 720 min**	ZnO_Intra	ZnO_Chem	1.02 x 10^{-4}
	ZnO_Phyto		2.40 x 10^{-5}
	ZnO_Extra		1.14 x 10^{-5}
	ZnO_Prot		1.18 x 10^{-2}

Table S3 The Dunnett post hoc test for DPPH assay. Cross correlation between each type of functionalized ZnO NPs in comparison to the bare ZnO NPs. The statistically significant correlations ($p < 0.01$) are marked in the red colour

Dependent variable	Tested samples		Significance (p-value)
**c = 1.56 µg/mL**	ZnO_Intra	ZnO_Chem	1..10 x 10^{-6}
	ZnO_Phyto		1.84 x 10^{-1}
	ZnO_Extra		1.88 x 10^{-4}
	ZnO_Prot		1.59 x 10^{-3}
**c = 3.12 µg/mL**	ZnO_Intra	ZnO_Chem	7.87 x 10^{-7}
	ZnO_Phyto		9.96 x 10^{-1}
	ZnO_Extra		1.08 x 10^{-5}
	ZnO_Prot		6.94 x 10^{-5}
**c = 6.25 µg/mL**	ZnO_Intra	ZnO_Chem	4.45 x 10^{-5}
	ZnO_Phyto		9.77 x 10^{-1}
	ZnO_Extra		1.60 x 10^{-5}
	ZnO_Prot		6.15 x 10^{-3}
**c = 12.5µg/mL**	ZnO_Intra	ZnO_Chem	9.95 x 10^{-1}
	ZnO_Phyto		3.93 x 10^{-2}
	ZnO_Extra		3.09 x 10^{-1}
	ZnO_Prot		2.70 x 10^{-5}
**c = 25 µg/mL**	ZnO_Intra	ZnO_Chem	4.42 x 10^{-1}
	ZnO_Phyto		9.98 x 10^{-1}
	ZnO_Extra		3.02 x 10^{-1}
	ZnO_Prot		5.54 x 10^{-6}
**c = 50 µg/mL**	ZnO_Intra	ZnO_Chem	5.17 x 10^{-3}
	ZnO_Phyto		9.63 x 10^{-1}
	ZnO_Extra		2.86 x 10^{-3}
	ZnO_Prot		1.14 x 10^{-6}
**c = 100 µg/mL**	ZnO_Intra	ZnO_Chem	4.84 x 10^{-2}
	ZnO_Phyto		1.91 x 10^{-1}
	ZnO_Extra		2.95 x 10^{-1}
	ZnO_Prot		4.18 x 10^{-6}
**c = 200 µg/mL**	ZnO_Intra	ZnO_Chem	9.97 x 10^{-1}
	ZnO_Phyto		5.02 x 10^{-2}
	ZnO_Extra		6.67 x 10^{-1}
	ZnO_Prot		3.43 x 10^{-4}

Table S4 The Dunnett post hoc test for MTT viability assay. Cross correlation between each type of functionalized ZnO NPs in comparison to the bare ZnO NPs. The statistically significant correlations ($p < 0.01$) are marked in the red colour

Dependent variable	Tested samples		Significance (p-value)	
			**Caco-2 cells**	**L929 cells**
**c = 1.56 μg/mL**	ZnO_Intra	ZnO_Chem	7.7×10^{-2}	8.86×10^{-4}
	ZnO_Phyto		3.09×10^{-2}	7.56×10^{-1}
	ZnO_Extra		2.01×10^{-2}	9.85×10^{-1}
	ZnO_Prot		1.77×10^{-6}	9.8×10^{-1}
**c = 3.12 μg/mL**	ZnO_Intra	ZnO_Chem	1.43×10^{-1}	2.46×10^{-6}
	ZnO_Phyto		2.09×10^{-2}	8.58×10^{-6}
	ZnO_Extra		3.44×10^{-1}	2.17×10^{-1}
	ZnO_Prot		6.49×10^{-4}	3.64×10^{-1}
**c = 6.25 μg/mL**	ZnO_Intra	ZnO_Chem	2.72×10^{-2}	3.06×10^{-3}
	ZnO_Phyto		4.24×10^{-2}	2.87×10^{-3}
	ZnO_Extra		7.66×10^{-1}	2.58×10^{-1}
	ZnO_Prot		2.07×10^{-5}	8.94×10^{-1}
**c = 12.5μg/mL**	ZnO_Intra	ZnO_Chem	7.85×10^{-2}	1.96×10^{-4}
	ZnO_Phyto		1.33×10^{-1}	1.94×10^{-4}
	ZnO_Extra		2.58×10^{-1}	2.19×10^{-4}
	ZnO_Prot		1.44×10^{-4}	6.77×10^{-1}
**c = 25 μg/mL**	ZnO_Intra	ZnO_Chem	6.39×10^{-5}	3.12×10^{-8}
	ZnO_Phyto		5.85×10^{-4}	3.12×10^{-8}
	ZnO_Extra		8.51×10^{-4}	3.12×10^{-8}
	ZnO_Prot		5.32×10^{-8}	1.12×10^{-3}
**c = 50 μg/mL**	ZnO_Intra	ZnO_Chem	3.28×10^{-4}	4.22×10^{-1}
	ZnO_Phyto		5.43×10^{-3}	3.82×10^{-1}
	ZnO_Extra		8.95×10^{-4}	1
	ZnO_Prot		3.58×10^{-8}	9.66×10^{-1}
**c = 100 μg/mL**	ZnO_Intra	ZnO_Chem	1	9.98×10^{-1}
	ZnO_Phyto		2.21×10^{-2}	6.84×10^{-2}
	ZnO_Extra		1.38×10^{-2}	1.87×10^{-1}
	ZnO_Prot		3.64×10^{-3}	9.92×10^{-1}
**c = 200 μg/mL**	ZnO_Intra	ZnO_Chem	5.00×10^{-1}	8.92×10^{-3}
	ZnO_Phyto		1.52×10^{-3}	2.72×10^{-4}
	ZnO_Extra		2.04×10^{-3}	4.38×10^{-3}
	ZnO_Prot		3.55×10^{-2}	1

Table S5 The Dunnett post hoc test for LDH membrane integrity assay. Cross correlation between each type of functionalized ZnO NPs in comparison to the bare ZnO NPs. The statistically significant correlations ($p < 0.01$) are marked in the red colour

Dependent variable	Tested samples		Significance (p-value)	
			**Caco-2 cells**	**L929 cells**
**c = 1.56 μg/mL**	ZnO_Intra	ZnO_Chem	7.39×10^{-2}	3.5×10^{-8}
	ZnO_Phyto		4.92×10^{-1}	6.28×10^{-1}
	ZnO_Extra		7.12×10^{-1}	1.84×10^{-2}
	ZnO_Prot		4.51×10^{-1}	9.74×10^{-1}
**c = 3.12 μg/mL**	ZnO_Intra	ZnO_Chem	1.7×10^{-6}	3.21×10^{-8}
	ZnO_Phyto		6.99×10^{-1}	9.62×10^{-1}
	ZnO_Extra		5.11×10^{-1}	3.21×10^{-8}

	ZnO_Prot		4.86×10^{-1}	1
**c = 6.25 µg/mL**	ZnO_Intra	ZnO_Chem	3.22×10^{-8}	3.21×10^{-8}
	ZnO_Phyto		6.94×10^{-3}	3.21×10^{-8}
	ZnO_Extra		3.21×10^{-8}	3.21×10^{-8}
	ZnO_Prot		8.45×10^{-1}	9.51×10^{-1}
**c = 12.5µg/mL**	ZnO_Intra	ZnO_Chem	6.72×10^{-1}	3.21×10^{-8}
	ZnO_Phyto		1.34×10^{-6}	3.21×10^{-8}
	ZnO_Extra		9.29×10^{-1}	3.21×10^{-8}
	ZnO_Prot		1	1
**c = 25 µg/mL**	ZnO_Intra	ZnO_Chem	6.33×10^{-1}	4.71×10^{-6}
	ZnO_Phyto		2.07×10^{-4}	3.38×10^{-8}
	ZnO_Extra		7.86×10^{-5}	3.24×10^{-8}
	ZnO_Prot		6.76×10^{-1}	2.03×10^{-1}
**c = 50 µg/mL**	ZnO_Intra	ZnO_Chem	1.39×10^{-3}	3.51×10^{-8}
	ZnO_Phyto		7.83×10^{-4}	1.91×10^{-2}
	ZnO_Extra		3.46×10^{-4}	$6.42.66 \times 10^{-1}$
	ZnO_Prot		1	9.97×10^{-1}
**c = 100 µg/mL**	ZnO_Intra	ZnO_Chem	3.25×10^{-8}	8.92×10^{-3}
	ZnO_Phyto		4.66×10^{-6}	3.21×10^{-8}
	ZnO_Extra		2.4×10^{-5}	5.58×10^{-5}
	ZnO_Prot		3.46×10^{-6}	9.48×10^{-1}
**c = 200 µg/mL**	ZnO_Intra	ZnO_Chem	3.38×10^{-8}	4.39×10^{-8}
	ZnO_Phyto		4.04×10^{-8}	9.23×10^{-8}
	ZnO_Extra		4.01×10^{-8}	2.59×10^{-3}
	ZnO_Pro		3.29×10^{-6}	8.27×10^{-3}

Table S6 The Dunnett post hoc test for ROS generation assay. Cross correlation between each type of functionalized ZnO NPs in comparison to the bare ZnO NPs. The statistically significant correlations ($p < 0.01$) are marked in the red colour

Dependent variable	Tested samples		Significance (p-value) Caco-2	Significance (p-value) L929
**c = 1.56 µg/mL**	ZnO_Intra	ZnO_Chem	3.24×10^{-8}	3.77×10^{-5}
	ZnO_Phyto		3.75×10^{-8}	2.96×10^{-5}
	ZnO_Extra		6.31×10^{-8}	7.95×10^{-5}
	ZnO_Prot		3.71×10^{-1}	9.99×10^{-1}
**c = 3.12 µg/mL**	ZnO_Intra	ZnO_Chem	3.92×10^{-8}	3.64×10^{-8}
	ZnO_Phyto		1.02×10^{-7}	4.40×10^{-8}
	ZnO_Extra		5.07×10^{-8}	4.34×10^{-8}
	ZnO_Prot		4.63×10^{-3}	6.98×10^{-5}
**c = 6.25 µg/mL**	ZnO_Intra	ZnO_Chem	3.22×10^{-8}	5.58×10^{-7}
	ZnO_Phyto		3.22×10^{-8}	3.45×10^{-7}
	ZnO_Extra		3.22×10^{-8}	1.41×10^{-6}
	ZnO_Prot		3.22×10^{-8}	7.42×10^{-6}
**c = 12.5µg/mL**	ZnO_Intra	ZnO_Chem	6.12×10^{-8}	1.53×10^{-5}
	ZnO_Phyto		5.95×10^{-8}	4.39×10^{-5}
	ZnO_Extra		5.14×10^{-8}	1.25×10^{-4}
	ZnO_Prot		8.87×10^{-8}	5.50×10^{-5}
**c = 25 µg/mL**	ZnO_Intra	ZnO_Chem	2.31×10^{-1}	7.61×10^{-4}
	ZnO_Phyto		5.78×10^{-1}	5.11×10^{-5}
	ZnO_Extra		1.04×10^{-1}	2.17×10^{-4}
	ZnO_Prot		5.62×10^{-2}	2.12×10^{-1}
**c = 50 µg/mL**	ZnO_Intra	ZnO_Chem	1.10×10^{-1}	8.33×10^{-1}

	ZnO_Phyto		3.76 x 10^{-1}	9.69 x 10^{-1}
	ZnO_Extra		4.58 x 10^{-2}	3.73 x 10^{-1}
	ZnO_Prot		3.32 x 10^{-1}	2.04 x 10^{-1}
**c = 100 µg/mL**	ZnO_Intra	ZnO_Chem	1.22 x 10^{-1}	1.54 x 10^{-4}
	ZnO_Phyto		9.76 x 10^{-1}	1.04 x 10^{-2}
	ZnO_Extra		2.67 x 10^{-1}	1.28 x 10^{-1}
	ZnO_Prot		6.44 x 10^{-2}	8.04 x 10^{-1}
**c = 200 µg/mL**	ZnO_Intra	ZnO_Chem	1.42 x 10^{-4}	9.85 x 10^{-7}
	ZnO_Phyto		1.48 x 10^{-2}	5.39 x 10^{-5}
	ZnO_Extra		5.95 x 10^{-3}	8.10 x 10^{-7}
	ZnO_Pro		4.91 x 10^{-1}	4.85 x 10^{-1}
